# A Personalized Management Approach of OHSS: Development of a Multiphase Prediction Model and Smartphone-Based App

**DOI:** 10.3389/fendo.2022.911225

**Published:** 2022-07-06

**Authors:** Mingzhu Cao, Zhi Liu, Yanshan Lin, Yiqun Luo, Sichen Li, Qing Huang, Haiying Liu, Jianqiao Liu

**Affiliations:** ^1^ Department of Obstetrics and Gynecology, Center for Reproductive Medicine, Key Laboratory for Major Obstetric Diseases of Guangdong Province, The Third Affiliated Hospital of Guangzhou Medical University, Guangzhou, China; ^2^ Key Laboratory for Reproductive Medicine of Guangdong Province, The Third Affiliated Hospital of Guangzhou Medical University, Guangzhou, China; ^3^ Department of Ultrasound, Nanfang Hospital, Southern Medical University, Guangzhou, China

**Keywords:** multi-phase prediction models, bid data, smartphone-based app, risk calculation, self-monitor

## Abstract

**Objective:**

This study aimed to develop multiphase big-data-based prediction models of ovarian hyperstimulation syndrome (OHSS) and a smartphone app for risk calculation and patients’ self-monitoring.

**Methods:**

Multiphase prediction models were developed from a retrospective cohort database of 21,566 women from January 2017 to December 2020 with controlled ovarian stimulation (COS). There were 17,445 women included in the final data analysis. Women were randomly assigned to either training cohort (n = 12,211) or validation cohort (n = 5,234). Their baseline clinical characteristics, COS-related characteristics, and embryo information were evaluated. The prediction models were divided into four phases: 1) prior to COS, 2) on the day of ovulation trigger, 3) after oocyte retrieval, and 4) prior to embryo transfer. The multiphase prediction models were built with stepwise regression and confirmed with LASSO regression. Internal validations were performed using the validation cohort and were assessed by discrimination and calibration, as well as clinical decision curves. A smartphone-based app “OHSS monitor” was constructed as part of the built-in app of the IVF-aid platform. The app had three modules, risk prediction module, symptom monitoring module, and treatment monitoring module.

**Results:**

The multiphase prediction models were developed with acceptable distinguishing ability to identify OHSS at-risk patients. The C-statistics of the first, second, third, and fourth phases in the training cohort were 0.628 (95% CI 0.598–0.658), 0.715 (95% CI 0.688–0.742), 0.792 (95% CI 0.770–0.815), and 0.814 (95% CI 0.793–0.834), respectively. The calibration plot showed the agreement of predictive and observed risks of OHSS, especially at the third- and fourth-phase prediction models in both training and validation cohorts. The net clinical benefits of the multiphase prediction models were also confirmed with a clinical decision curve. A smartphone-based app was constructed as a risk calculator based on the multiphase prediction models, and also as a self-monitoring tool for patients at risk.

**Conclusions:**

We have built multiphase prediction models based on big data and constructed a user-friendly smartphone-based app for the personalized management of women at risk of moderate/severe OHSS. The multiphase prediction models and user-friendly app can be readily used in clinical practice for clinical decision-support and self-management of patients.

## Introduction

Ovarian hyperstimulation syndrome (OHSS) is a common and severe iatrogenic complication of ovarian hyperstimulation with an incidence of 2% to 6% of moderate OHSS and 0.1% to 0.2% of severe OHSS (1). OHSS is a self-limiting condition, and the symptoms will alleviate shortly. A small proportion of women with moderate/severe OHSS might have persistent discomforts, especially those with late-onset OHSS who will have extended symptoms till early pregnancy.

A variety of measures have been proposed to be useful in the prevention of OHSS, including decreased gonadotropin consumption, GnRH antagonist protocol for controlled ovarian hyperstimulation (COS), and GnRH agonist (GnRHa) for trigger and cryopreservation of all embryos (2). Despite the increasing methods of preventing OHSS, moderate/severe OHSS still occurred on a worldwide scale (3). Complete prevention of OHSS seems to be impossible. Although rare, the mortality risk can happen in around 1 in 450,000 to 500, 000 women (4). Therefore, an early prediction and prevention of OHSS is critical to reducing the morbidity of OHSS.

One challenge for the management of OHSS is to determine what clinical features may predispose the patient to an increased risk of OHSS. Although several prediction models of OHSS had been proposed, the overall predictive value of the possible influential factors of OHSS has not been well studied. Besides, an easy-to-use tool for clinicians to predict OHSS is not available. Moreover, for patients, lack of prompt and proper diagnosis of OHSS is a common problem. Thus, self-monitoring of OHSS-related symptoms and early detection of OHSS are particularly important for those women.

Here, we established big-data-based multiphase prediction models of OHSS. Furthermore, a user-friendly smartphone-based app was built based on the multiphase prediction models to assist clinicians in decision-making and help patients for self-monitoring. The final purpose of this study is to build a user-friendly tool to aid personalized management of OHSS.

## Materials and Methods

### Study Population

This was a retrospective cohort study, and the study protocol was in accordance with the Transparent Reporting of a Multivariable Prediction Model for Individual Prognosis or Diagnosis (TRIPOD) statement (5).

Data were obtained from the database in a locally largest reproductive center from January 2017 to December 2020. Infertile women attempting for *in vitro* fertilization (IVF) or intra-cytoplasmic sperm injection (ICSI) treatment and who underwent COS were included. Each couple had a unique record number for their medical record, which was saved at a local database. Every couple has signed an informed consent for using their medical record for data analysis. The study protocol was reviewed and approved by the hospital ethical committee (approval number, 2021-117).

**Figure 1:**
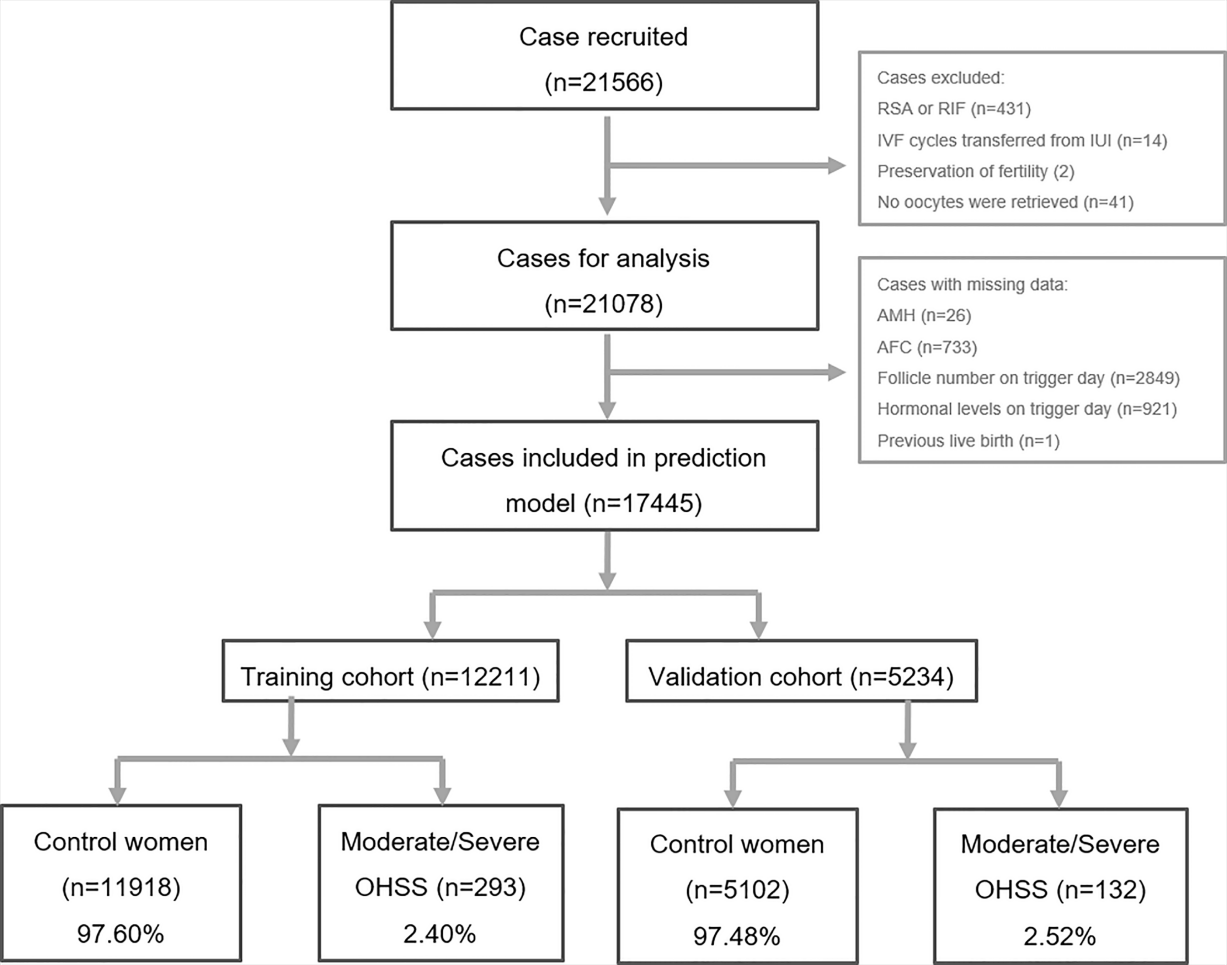
Flowchart of patients’ data inclusion and exclusion. RSA, repeated spontaneous abortion; RIF, repeated implantation failure; IVF, *in vitro* fertilization; IUI, intrauterine insemination; AMH, anti-Mullerian hormone; AFC, antral follicle counting; OHSS, ovarian hyper-stimulation syndrome.

Data of women who fulfilled the following criteria were included: 1) women aged 20–40 years old and 2) ovarian stimulation with GnRH agonist protocol or GnRH antagonist protocol. There were 21,566 cases included. Then, cases that met the following criteria were excluded: 1) women with recurrent spontaneous miscarriage or recurrent implantation failure (n = 431), 2) IVF cycles transferred from intrauterine insemination cycles (n = 14), 3) preservation of fertility due to malignancies (n = 2), and 4) no oocytes were retrieved (n = 41). Therefore, 21,078 cases were collected for further analysis. Cases with missing data (see **Figure 1** for details) were further excluded, and hence a total of 17,445 COS cycles were finally included for the development of the prediction model. Data from 17,445 cycles were randomly assigned to either the training cohort or the validation cohort at a ratio of 5:1.

### Ovarian Stimulation Procedures and Embryo Transfer

Two controlled ovarian stimulation protocols were used in the present study, the GnRH agonist protocol (62.1% in the training cohort and 61.7% in the validation cohort) and the GnRH antagonist protocol (37.9% in the training cohort and 38.3% in the validation cohort). Initial doses of 100 to 300 IU/day of recombinant FSH (Gonal-F, Merck, Switzerland; Puregon, Organon, Netherlands; and urofollitropin, Livzon Group, Zhuhai, China) and/or human menopausal gonadotropin (HMG, Livzon Group, China) were administrated. The protocols were chosen based on patients’ individual conditions (including age, ovarian reservation, weight, previous ovarian stimulation response) and physicians’ preferences. Growth of ovarian follicles and serum follicular-stimulating hormone (FSH), luteinizing hormone (LH), estradiol (E2), and progesterone (P) levels was monitored on a regular basis. The gonadotropin (Gn) doses were adjusted according to the development of follicles and serum hormone levels. Triggering of ovulation was provided if the patients had at least three follicles reaching 17 mm or at least two follicles reaching 18 mm in diameter. Transvaginal oocyte retrieval was scheduled 34 to 36 h following the ovulation triggering with HCG (Ovidrel, Merck, Switzerland; HCG, Livzon Group, China) and/or GnRH agonist (triptorelin acetate, Ferring, Kiel, Germany) injection.

Oocytes were collected with a standard oocyte retrieval procedure under the real-time monitoring of transvaginal sonography. Either IVF or ICSI was provided based on semen quality. Fertilization was observed by the appearance of two pronuclei. Day 3 cleavage embryos were monitored and graded. Fresh embryo transfer was scheduled with cleavage embryo on day 3 or blastocysts on day 5 or 6 following oocyte collection. The remaining embryos or embryos in freeze-all strategies were vitrified as cleavage embryos or blastocysts.

In our center, the freeze-all decision was made based on either one of the following conditions: 1) the number of oocytes retrieved ≥20; 2) the serum level of E2 ≥18,350 pmol/L on trigger day; 3) impaired endometrial receptivity induced by endometrial polyps, submucosal fibroid, intrauterine cavity fluid, untreated hydrosalpinx, etc.; 4) other medical conditions not suitable for fresh embryo transfer as determined by physicians; and 5) patient’s personal reasons who cannot arrange fresh embryo transfer.

### Outcomes Measured

The primary outcome was the occurrence of moderate/severe OHSS. The diagnosis of moderate/severe OHSS was based on the criteria as suggested by a consensus of Chinese experts (1). The criteria for moderate OHSS are as follows: 1) the patient presents with abdominal distention, nausea, vomit, or diarrhea; 2) sonography exam shows ovarian enlargement to 8–12 cm and presence of ascites; 3) laboratory test shows hematocrit <0.45, elevated leukocyte (10–15 × 10^9^/l). The criteria for severe OHSS are as follows: 1) patient presents with severe nausea, vomit, dyspnea, notable abdominal pain, oliguria, or even anuria (<300 ml/day or <30 ml/h), rapid increase of body weight (>1 kg/24 h); 2) sonography exam shows ovarian enlargement to >12 cm, presence of tension ascites, pleural effusion, vascular embolism, low blood pressure, or low central venous pressure; and 3) laboratory test shows increased hematocrit (>0.45), elevated leukocyte (>15 × 10^9^/l), hyperkalemia (potassium >5 mmol/l), hyponatremia (sodium < 135 mmol/l), damaged renal function (creatine > 1.0 g/l), and damaged liver function (with increased levels of glutamic oxaloacetic transaminase and glutamic pyruvic transaminase). Women who were suspected to develop OHSS were made an appointment with one specialist. Detailed symptoms, physical examination, transabdominal ultrasound, and laboratory tests were recorded in the medical database. Patients who were confirmed with OHSS were administrated aspirin, letrozole, intravenous albumin, or low molecular heparin, as appropriate.

### Variables Evaluated in the Models

Potential variables which might be included in the prediction model were prespecified generally based on clinical experience and literature reports. Basic clinical characteristics including female age, infertile duration, infertile type, infertile factors, height, weight, body mass index (BMI), body surface area (equations estimating the body surface area for the Chinese population were derived from (6)), previous live birth, anti-Mullerian hormone (AMH), antra follicle counting (AFC), and diagnosis as polycystic ovarian syndrome (PCOS) were recorded and extracted from a local database. COS cycle-related parameters, including COS cycle number, COS protocol, Gn duration, initial dose and mean dose of Gn, total dose of Gn, number of follicles with various diameters on trigger day, hormonal levels on trigger day (LH, P, and E2), type of trigger (including HCG alone, GnRHa alone, and dual trigger), number of oocytes retrieved, freeze-all strategy, fertilization type, and origin and type of sperms, were obtained. For those with fresh embryo transfer (ET), the number of cycles with ET, number of embryos for ET, number of top-quality embryos for ET, and number of blastocysts for ET were also recorded.

### Statistical Analysis

#### General Statistical Descriptions and Analysis

All data analyses were conducted using SPSS (version 22.0, IBM Corp., Chicago, Illinois, USA) and R package (version 4.1.2, Vienna, Austria). Continuous data following normal distribution were described as mean ± standard deviation (SD) and were compared using Student’s t-test. Continuous data which were not following the normal distribution were described as median and 25th and 75th percentiles and were compared using the Wilcoxon rank-sum test. Categorical data were described as frequency and percentage and were compared using the chi-square test. A *P*-value < 0.05 was considered statistically significant. All eligible patients were randomized at a 7:3 ratio to create a developmental (training) cohort and a validation cohort, respectively.

#### Development of the Prediction Models

The multiphase prediction models were developed based on the developmental (training) cohort at the following point times of critical decision-making: 1) prior to COS, 2) prior to ovulation trigger, 3) on the day of oocyte retrieval, and 4) on the day of fresh embryo transfer. The multistage prediction models were made based on variables at corresponding stages. The selection of variables in the above phases was based on the stepwise regression models and confirmed with LASSO regression which demonstrated to have similar performances of prediction. The final models were fitted using stepwise backward selection and presented as odds ratio (OR) and 95% confidential index (95% CI).

Multiphase nomograms with graphic details to predict moderate/severe OHSS in COS cycles were also generated. Values for each covariate were mapped to the points on a special scale ranging from zero to 100. A total point obtained from each covariate was summed up and corresponded to the possibilities of OHSS.

#### Validation and Evaluation of the Prediction Model

Internal validations of the prediction models were performed using the validation cohort and were assessed by both discrimination and calibration. The discrimination ability of the models was demonstrated in areas under the curve (AUC, or C-statistic) of the receiver operating characteristic (ROC) curves. A higher C-statistic close to 1 refers to a perfect prediction. Generally, C-statistics above 0.7 are acceptable in clinical settings (7).

Calibration of the multistage prediction models was evaluated by calibration plot and Hosmer–Lemeshow good-of-fit test, which was plotted to compare the predicted probability of OHSS and the actual probability of OHSS.

Clinical decision curves were plotted to evaluate the clinical utility of the multiphase models. The clinical benefit was determined by the net benefit. The value zero represents no benefit, whereas higher values represent more benefit.

#### Construction of the Smartphone-Based App

The app was constructed as part of the built-in app of the IVF-aid platform, which was developed by our Reproductive Medical Center (software copyright registration number, 7857265). The self-monitoring part of the app, which was named as “OHSS monitor,” has three modules, risk prediction, symptoms monitoring, and treatment monitoring. 1) The “risk prediction module” was used to calculate the individual risk of developing OHSS based on the multiphase of the prediction models. The first three phases of risk prediction can only be accessed by clinicians for assisting clinical decision, and the final phase of risk prediction results will be open to the at-risk patients for OHSS risk alert. All variables of the prediction model could be extracted from the IVF-aid platform with additional data input, and an automatic calculation of OHSS risk would be provided to the high-risk individual. 2) The “symptom monitoring module” includes most frequent discomforts related to OHSS, and the severity of symptoms was further divided into four ranks revised from the Likert scale (8). 3) The “treatment monitoring module” records an individual’s visit to the hospital and their personal medical chart for the details of treatment.

## Results

### Basic Clinical Characteristics of Patients in the Training and Validation Cohorts

In the present study, 21,566 cases were recorded and scrutinized as demonstrated in **Figure 1**. After the exclusion of women with missing data (see **Figure 1** for details), 17,445 cases with sufficient information were recruited in the final analysis. Among them, 12,211 cases (70.0%) were randomly picked as the training cohort, and the remaining 5,234 cases (30.0%) were selected as the validation cohort. There were 293 patients (2.40%) determined to have moderate/severe OHSS in the training cohort, and 132 (2.52%) in the validation cohort were diagnosed with moderate/severe OHSS. Those women with moderate/severe OHSS were termed as “moderate/severe OHSS group,” and the remaining cases were termed as “control group.”

As demonstrated in **Tables 1**, **2**, the basic clinical characteristics and COS cycle-related characteristics showed no differences between the training cohort and validation cohort. As expected, women in the moderate/severe OHSS group showed decreased age, BMI, body surface area, increased ovarian reservation markers (AMH, AFC, PCOS), and more primary infertility (**Table 1**). The majority of women with moderate/severe OHSS (88.74%% in the training cohort and 88.64% in the validation cohort) were in their first COS cycle. COS protocols (either GnRH agonist protocol or GnRH antagonist protocol) were comparable between the two groups. However, women with moderate/severe OHSS had decreased doses of Gn but increased number of follicles on trigger day no matter what size of follicles. Besides, women in the moderate/severe OHSS group presented with elevated levels of estradiol on trigger day and had more oocytes retrieved. More of them had HCG alone for trigger, and less of them had all embryos frozen, hence more women with fresh embryo transfer (see **Table 2** for details).

**Table 1:** Baseline clinical characteristics of the training and validation cohorts.

	Training cohort				Validation cohort				Training vs. validation
	Control	Moderate/severe OHSS	Total	*P* value	Control	Moderate/severe OHSS	Total	*P* value	*P* value
Female age (years old)	31.88 ± 4.45	30.59 ± 3.67	31.85 ± 4.44	<.001	31.86 ± 4.43	30.05 ± 3.65	31.81 ± 4.42	<.001	0.596
<35	8,481 (71.2%)	253 (86.3%)	8,734 (71.5%)	<.001	3,646 (71.5%)	114 (86.4%)	3,760 (71.8%)	<.001	0.675
≥35	3,437 (28.8%)	40 (13.7%)	3,477 (28.5%)		1,456 (28.5%)	18 (13.6%)	1,474 (28.2%)		
Infertile duration (years)	4 (2–6)	4 (2–5)	4 (2–6)	0.357	4 (2–6)	4 (2–5.75)	4 (2–6)	0.246	0.612
Infertile type									
Primary infertility	5,934 (49.8%)	168 (57.3%)	6,102 (50.0%)	0.011	2,478 (48.6%)	76 (57.6%)	2,554 (48.8%)	0.041	0.155
Secondary infertility	5,984 (50.2%)	125 (42.7%)	6,109 (50.0%)		2,624 (51.4%)	56 (42.4%)	2,680 (51.2%)		
Infertile factors									
Tubal/pelvic factor	5,412 (45.4%)	122 (41.6%)	5,534 (45.3%)	0.188	2,316 (45.4%)	56 (42.4%)	2,372 (45.3%)	0.82	0.331
Ovulation disorder	846 (7.1%)	30 (10.2%)	876 (7.2%)		341 (6.7%)	9 (6.8%)	350 (6.7%)		
Endometriosis	261 (2.2%)	4 (1.4%)	265 (2.2%)		93 (1.8%)	4 (3.0%)	97 (1.9%)		
Male factor	1,716 (14.4%)	48 (16.4%)	1,764 (14.4%)		791 (15.5%)	19 (14.4%)	810 (15.5%)		
Mixed factors	2,695 (22.6%)	61 (20.8%)	2,756 (22.6%)		1,138 (22.3%)	30 (22.7%)	1,168 (22.3%)		
Unexplained	988 (8.3%)	28 (9.6%)	1,016 (8.3%)		423 (8.3%)	14 (10.6%)	437 (8.3%)		
Height (m)	157.91 ± 5.07	158.37 ± 5.02	157.92 ± 5.07	0.126	157.82 ± 5.13	157.87 ± 4.88	157.83 ± 5.13	0.916	0.275
Weight (kg)	54.73 ± 8.34	53.79 ± 7.47	54.71 ± 8.32	0.035	54.85 ± 8.29	52.88 ± 6.98	54.80 ± 8.27	0.002	0.513
BMI (kg/m^2^)	21.94 ± 3.14	21.44 ± 2.76	21.93 ± 3.13	0.003	22.01 ± 3.13	21.21 ± 2.62	21.99 ± 3.12	<.001	0.215
Body surface area	1.50 (1.43–1.58)	1.49 (1.43–1.56)	1.50 (1.43–1.58)	0.161	1.50 (1.43–1.58)	1.48 (1.41–1.56)	1.50 (1.43–1.58)	0.008	0.767
AMH (ng/mL)	5.29 ± 3.87	6.46 ± 4.10	5.32 ± 3.88	<.001	5.21 ± 3.89	7.20 ± 4.12	5.26 ± 3.91	<.001	0.166
Basal FSH level (IU/L)	5.60 ± 1.70	5.39 ± 1.41	5.60 ± 1.69	0.052	5.58 ± 1.69	5.24 ± 1.46	5.57 ± 1.68	0.027	0.274
AFC (n)	19.09 ± 8.66	21.06 ± 8.26	19.13 ± 8.66	<.001	19.01 ± 8.48	23.10 ± 7.38	19.11 ± 8.48	<.001	0.959
Previous live birth									
n = 0	8,970 (75.3%)	252 (86.0%)	9,222 (75.5%)	<.001	3,804 (74.6%)	115 (87.1%)	3,919 (74.9%)	0.013	0.636
n = 1	2,791 (23.4%)	39 (13.3%)	2,830 (23.2%)		1,220 (23.9%)	16 (12.1%)	1,236 (23.6%)		
n = 2	148 (1.2%)	2 (0.7%)	150 (1.2%)		73 (1.4%)	1 (0.8%)	74 (1.4%)		
n = 3	9 (0.1%)	0 (0.0%)	9 (0.1%)		5 (0.1%)	0 (0.0%)	5 (0.1%)		
PCOS									
No	9,897 (83.0%)	222 (75.8%)	10,119 (82.9%)	0.001	4,257 (83.4%)	102 (77.3%)	4,359 (83.3%)	0.061	0.504
Yes	2,021 (17.0%)	71 (24.2%)	2,092 (17.1%)		845 (16.6%)	30 (22.7%)	875 (16.7%)		

OHSS, ovarian hyper-stimulation syndrome; BMI, body mass index; AMH, anti-Mullerian hormone; FSH, follicular stimulating hormone; AFC, antral follicle counting; PCOS, polycystic ovarian syndrome.

**Table 2:** Controlled ovarian stimulation cycle related characteristics of the training and validation cohorts.

	Training cohort				Validation cohort				Training vs. validation
	Controln = 11,918	Moderate/severe OHSS n = 293	Totaln = 12211	*P* value	Controln = 5102	Moderate/severe OHSS n = 132	Totaln = 5234	*P* value	*P* value
COS cycle number	1 (1–1)	1 (1–1)		0.003	1 (1–1)	1 (1–1)	1 (1–1)	0.027	0.339
First cycle	9,621 (80.73%)	260 (88.74%)	9,881 (80.92%)		4,085 (80.07%)	117 (88.64%)	4,202 (80.28%)		
Second cycle	2,052 (17.22%)	32 (10.92%)	2,084 (17.07%)		910 (17.84%)	14 (10.61%)	924 (17.65%)		
COS protocol									0.605
GnRH agonist protocol	7,399 (62.1%)	185 (63.1%)	7,584 (62.1%)	0.712	3,148 (61.7%)	81 (61.4%)	3,229 (61.7%)	0.937	0.605
GnRH antagonist protocol	4,519 (37.9%)	108 (36.9%)	4,627 (37.9%)		1,954 (38.3%)	51 (38.6%)	2,005 (38.3%)		
Gn duration	11.15 ± 2.22	10.79 ± 1.94	11.14 ± 2.22	0.012	11.11 ± 2.23	11.09 ± 2.27	11.11 ± 2.24	0.952	0.145
Total dose of Gn (IU)	2,025 (1,500–2,775)	1650 (1,231.25–2,175)	2025 (1,500–2,737.5)	<.001	2,025 (1,500–2,812.5)	1637 (1,200–2,090.63)	2,025 (1,500–2,775)	<.001	0.466
Mean dose of Gn (IU)	187.5 (150–225)	150 (125–187.5)	187.5 (150–225)	<.001	187.5 (150–230)	150 (125–187.5)	187.5 (150–230)	<.001	0.062
Initial dose of Gn (IU)	150 (150–225)	150 (112.5–150)	150 (150–225)	<.001	150 (150–225)	150 (112.5–150)	150 (150–225)	<.001	0.025
Number of follicles on trigger day									
Follicles ≥ 10 mm	17 (11–24)	19 (14–26)	17 (11–24)	<.001	17 (11–24)	20 (15–32)	17 (11–24)	<.001	0.543
Follicles ≥ 14 mm	10 (7–13)	11 (9–14)	10 (7–13)	<.001	10 (7–13)	12 (9–16.5)	10 (7–13)	<.001	0.828
Follicles ≥ 16 mm	7 (5–9)	8 (6–10)	7 (5–9)	<.001	7 (5–9)	7.5 (6–10)	7 (5–9)	0.002	0.669
Follicles ≥ 18 mm	4 (2–5)	4 (3–6)	4 (2–5)	0.002	4 (2–5)	4 (2–5)	4 (2–5)	0.299	0.644
Hormonal levels on trigger day									
LH (IU/L)	1.33 ± 1.45	1.45 ± 1.42	1.33 ± 1.45	0.037	1.31 ± 1.15	1.25 ± 1.05	1.31 ± 1.15	0.29	0.445
P (nmol/L)	2.77 ± 1.98	2.43 ± 1.42	2.76 ± 1.97	<.001	2.79 ± 1.77	2.80 ± 2.07	2.79 ± 1.77	0.319	0.271
E2 (pmol/L)				0.038				0.006	0.796
E2 ≤ 1,835	69 (0.6%)	0 (0.0%)	69 (0.6%)		25 (0.5%)	0 (0.0%)	25 (0.5%)		
1,835 < E2 ≤ 3,670	408 (3.4%)	2 (0.7%)	410 (3.4%)		165 (3.2%)	0 (0.0%)	165 (3.2%)		
3,670 < E2 ≤ 18,350	8,895 (74.6%)	225 (76.8%)	9,120 (74.7%)		3,833 (75.1%)	90 (68.2%)	3,923 (75.0%)		
E2 > 18,350	2,546 (21.4%)	66 (22.5%)	2,612 (21.4%)		1,079 (21.1%)	42 (31.8%)	1,121 (21.4%)		
Type of trigger									
HCG alone	10,135 (85.0%)	284 (96.9%)	10,419 (85.3%)	<.001	4,313 (84.5%)	123 (93.2%)	4,436 (84.8%)	0.013	0.556
GnRHa alone	644 (5.4%)	1 (0.3%)	645 (5.3%)		294 (5.8%)	1 (0.8%)	295 (5.6%)		
Dual trigger	1,139 (9.6%)	8 (2.7%)	1,147 (9.4%)		495 (9.7%)	8 (6.1%)	503 (9.6%)		
Number of oocytes retrieved	13 (9–18)	15.5 (11–22)	13 (9–18)	<.001	13 (9–18)	15.5 (11–22)	13 (9–18)	<.001	0.779
≤10	4,218 (35.4%)	73 (24.9%)	4,291 (35.1%)	<.001	1,844 (36.1%)	30 (22.7%)	1,874 (35.8%)	<.001	0.594
11–20	5,637 (47.3%)	175 (59.7%)	5,812 (47.6%)		2,378 (46.6%)	61 (46.2%)	2,439 (46.6%)		
21–30	1,719 (14.4%)	32 (10.9%)	1,751 (14.3%)		727 (14.2%)	29 (22.0%)	756 (14.4%)		
> 30	344 (2.9%)	13 (4.4%)	357 (2.9%)		153 (3.0%)	12 (9.1%)	165 (3.2%)		
Freeze all									
No	7,469 (62.7%)	232 (79.2%)	7,701 (63.1%)	<.001	3,172 (62.2%)	89 (67.4%)	3,261 (62.3%)	0.219	0.340
Yes	4,449 (37.3%)	61 (20.8%)	4,510 (36.9%)		1,930 (37.8%)	43 (32.6%)	1,973 (37.7%)		
Fertilization type									
1 = IVF	9,786 (82.1%)	246 (84.0%)	10,032 (82.2%)	0.623	4,145 (81.2%)	108 (81.8%)	4,253 (81.3%)	0.951	0.178
2 = ICSI	2,118 (17.8%)	47 (16.0%)	2,165 (17.7%)		954 (18.7%)	24 (18.2%)	978 (18.7%)		
3 = IVF/ICSI	14 (0.1%)	0 (0.0%)	14 (0.1%)		3 (0.1%)	0 (0.0%)	3 (0.1%)		
Origin of sperm									
1 = husband	11,852 (99.4%)	290 (99.0%)	12,142 (99.4%)	0.289	5,071 (99.4%)	131 (99.2%)	5,202 (99.4%)	0.827	0.712
2 = sperm bank	66 (0.6%)	3 (1.0%)	69 (0.6%)		31 (0.6%)	1 (0.8%)	32 (0.6%)		
Type of sperm									
1 = fresh	11,799 (99.0%)	289 (98.6%)	12,088 (99.0%)	0.535	5,050 (99.0%)	131 (99.2%)	5,181 (99.0%)	0.767	0.974
2 = frozen	119 (1.0%)	4 (1.4%)	123 (1.0%)		52 (1.0%)	1 (0.8%)	53 (1.0%)		0.349
Number of cycles with ET	6,078 (51.0%)	216 (73.7%)	6,294 (51.5%)	<.001	2,574 (50.5%)	82 (62.1%)	2,656 (50.7%)	0.008	0.334
Number of cycles with canceled ET	5,840 (49.0%)	77 (26.3%)	5,917 (48.5%)		2,528 (49.5%)	50 (37.9%)	2,578 (49.3%)		
Number of embryos for ET	2 (1,2)	2 (2,2)	2 (1,2)	0.026	2 (1,2)	2 (2,2)	2 (1,2)	0.122	0.233
n = 1	1,902 (31.3%)	50 (23.1%)	1,952 (31.0%)	0.025	842 (32.7%)	19 (22.6%)	861 (32.4%)	0.059	0.399
n = 2	4,082 (67.2%)	164 (75.9%)	4,246 (67.5%)		1,689 (65.6%)	65 (77.4%)	1,754 (66.0%)		
n = 3	94 (1.5%)	2 (0.9%)	96 (1.5%)		43 (1.7%)	0 (0.0%)	43 (1.6%)		
Number of top-quality embryos for ET	1 (0,2)	1 (1,2)	1 (0,2)	<.001	1 (0,2)	2 (1,2)	1 (0,2)	0.001	0.506
n = 0	1,885 (31.0%)	45 (20.8%)	1,930 (30.7%)	<.001	747 (29.0%)	15 (18.3%)	762 (28.7%)	0.004	0.089
n = 1	2,161 (35.6%)	72 (33.0%)	2,233 (35.5%)		987 (38.3%)	25 (30.5%)	1,012 (38.1%)		
n = 2	2,006 (33.0%)	98 (45.4%)	2,104 (33.4%)		831 (32.3%)	42 (51.2%)	873 (32.9%)		
Number of blastocysts for ET	0 (0,0)	0 (0,0)	0 (0,0)	0.566	0 (0,0)	0 (0,0)	0 (0,0)	0.753	0.662
n = 0	5,265 (86.6%)	184 (85.2%)	5,449 (86.6%)	0.076	2,237 (86.9%)	72 (87.8%)	2,309 (86.9%)	0.341	0.778
n = 1	674 (11.1%)	31 (14.4%)	705 (11.2%)		275 (10.7%)	10 (12.2%)	285 (10.7%)		
n = 2	139 (2.3%)	1 (0.5%)	140 (2.2%)		62 (2.4%)	0 (0.0%)	62 (2.3%)		

OHSS, ovarian hyper-stimulation syndrome; COS, controlled ovarian stimulation; GnRH, gonadotropin-releasing hormone; Gn, gonadotropin; LH, luteinizing hormone; P, progesterone; E2, estradiol; HCG, human chorionic gonadotropin; GnRHa, gonadotropin-releasing hormone agonist; IVF, in vitro fertilization; ICSI, intra-cytoplasmic sperm injection; ET, embryo transfer.

### Development of multiphase prediction models

Variables included in different phases are presented in **Table 3**. Multiphase nomograms corresponding to the abovementioned phases were also constructed and are presented in **Supplementary Figure 1** (**Supplementary Figure 1A** for the first phase, **1B** for the second phase, **1C** for the third phase, and **1D** for the fourth phase prediction).

**Table 3:** Multivariable analysis of multiphase variables in predicting OHSS.

**Variables**	z value	*P* value	OR	95% CI-lower	95% CI-upper
**The 1st phase**					
Female age (years old)	-1.801	0.072	0.972	0.942	1.002
COS cycle number	-2.699	0.007	0.634	0.446	0.866
Previous live birth					
No			1.000	1.000	1.000
Yes	-2.521	0.012	0.631	0.436	0.893
AMH (ng/mL)	3.484	0.000	1.050	1.021	1.078
BMI (kg/m^2^)	-1.747	0.081	0.966	0.928	1.004
**The 2nd phase**					
Female age	1.519	0.129	1.028	0.992	1.065
COS cycle number	-2.101	0.036	0.701	0.492	0.958
Previous live birth					
No			1.000	1.000	1.000
Yes	-2.071	0.038	0.685	0.473	0.970
BMI (kg/m^2^)	0.656	0.512	1.014	0.971	1.059
AMH (ng/mL)	0.140	0.889	1.003	0.964	1.042
PCOS					
No			1.000	1.000	1.000
Yes	1.164	0.245	1.220	0.869	1.699
COS protocol					
GnRH agonist protocol			1.000	1.000	1.000
GnRH antagonist protocol	-4.587	0.000	0.447	0.316	0.629
Duration of Gn (days)	-4.217	0.000	0.853	0.792	0.917
Initial dose of Gn (IU)	-6.931	0.000	0.988	0.984	0.991
Number of follicles ≥ 16 mm on trigger day	3.476	0.001	1.069	1.029	1.109
E2 levels on trigger day (pmol/L)					
E2 < 3,670			1.000	1.000	1.000
3,670 ≤ E2 < 18,350	1.689	0.091	3.373	1.053	20.599
E2 ≥ 18,350	1.195	0.232	2.437	0.708	15.352
P levels on trigger day (nmol/L)	-1.972	0.049	0.912	0.829	0.993
**The 3rd phase**					
COS cycle number	-1.866	0.062	0.502	0.211	0.923
Previous live birth					
No			1.000	1.000	1.000
Yes	-1.643	0.100	0.748	0.523	1.046
AFC	-1.667	0.096	0.983	0.963	1.003
Body surface area	1.864	0.062	2.878	0.938	8.671
PCOS					
No			1.000	1.000	1.000
Yes	2.466	0.014	1.535	1.087	2.149
COS protocol					
GnRH agonist protocol			1.000	1.000	1.000
GnRH antagonist protocol	-1.496	0.135	0.763	0.535	1.085
Duration of Gn (days)	-3.209	0.001	0.873	0.802	0.947
Initial dose of Gn (IU)	-1.717	0.086	0.994	0.987	1.001
Mean dose of Gn (IU)	-1.793	0.073	0.993	0.986	1.001
Number of follicles ≥ 10 mm on trigger day	2.797	0.005	1.026	1.008	1.045
Number of follicles ≥ 18 mm on trigger day	3.461	0.001	1.099	1.041	1.159
E2 levels on trigger day (pmol/L)					
E2 <3,670			1.000	1.000	1.000
3,670 ≤ E2 < 18,350	1.270	0.204	2.500	0.777	15.298
E2 ≥ 18,350	1.840	0.066	4.019	1.135	25.650
Type of trigger					
Dual trigger			1.000	1.000	1.000
GnRHa alone	-2.004	0.045	0.118	0.006	0.652
HCG alone	5.233	0.000	7.322	3.690	16.712
Freeze all strategy					
No			1.000	1.000	1.000
Yes	-8.419	0.000	0.193	0.131	0.282
Number of oocytes retrieved	2.866	0.004	1.033	1.010	1.057
**The 4th phase**					
COS cycle number	-2.530	0.011	0.557	0.342	0.848
Previous live birth					
No			1.000	1.000	1.000
Yes	-1.706	0.088	0.739	0.515	1.035
AFC	-1.728	0.084	0.983	0.963	1.002
Body surface area	1.697	0.090	2.664	0.852	8.185
PCOS					
No			1.000	1.000	1.000
Yes	2.525	0.012	1.541	1.097	2.147
Duration of Gn (days)	-3.004	0.003	0.901	0.841	0.964
Initial dose of Gn (IU)	-3.004	0.003	0.901	0.841	0.964
Mean dose of Gn (IU)	-1.983	0.047	0.993	0.986	1.000
Number of follicles ≥ 16 mm on trigger day	4.286	0.000	1.093	1.049	1.138
E2 levels on trigger day (pmol/L)					
E2 <3,670			1.000	1.000	1.000
3,670 ≤ E2 < 18,350	0.948	0.343	1.983	0.615	12.145
E2 ≥ 18,350	2.031	0.042	4.703	1.307	30.255
Type of trigger					
Dual trigger			1.000	1.000	1.000
GnRHa alone	-1.941	0.052	0.126	0.007	0.699
HCG alone	5.118	0.000	6.884	3.499	15.628
Freeze all strategy					
No			1.000	1.000	1.000
Yes	-2.886	0.004	0.484	0.297	0.798
Number of oocytes retrieved	4.851	0.000	1.058	1.034	1.082
Number of cycles with ET	1.880	0.060	1.310	0.980	1.724
Number of embryo(s) for ET	3.611	0.000	1.685	1.270	2.239
Number of top-quality embryo(s) for ET	1.771	0.077	1.191	0.985	1.450

OHSS, ovarian hyper-stimulation syndrome; COS, controlled ovarian stimulation; AMH, anti-Mullerian hormone; BMI, body mass index; PCOS, polycystic ovarian syndrome; GnRH, gonadotropin-releasing hormone; Gn, gonadotropin; E2, estradiol; P, progesterone; GnRHa, gonadotropin releasing hormone agonist; HCG, human chorionic gonadotropin; ET, embryo transfer.

The equations of the multiphase prediction models are:

The 1st phase: P (OHSS) = 1 / (1 + exp(- (- 1.7165 - 0.0287 * Female age - 0.456 * Number of COS cycles - 0.4604 * Previous live birth (1) + 0.0484 *AMH - 0.035 * BMI))).
The 2nd phase: P (OHSS) = 1 / (1 + exp(- (- 1.7584 + 0.0276 * Female age - 0.3559 * Number of COS cycles - 0.3784 * Previous live birth (1) + 0.1989 * PCOS (yes) - 0.8059 * GnRH antagonist protocol - 0.1586 * Duration of Gn- 0.0126 * Initial dose of Gn + 0.0664 * Number of follicles (≥ 16 mm) on trigger day + 1.2158 * E2 levels on trigger day (3670 ≤ E2 < 18350 pmol/ml) + 0.8908 * E2 levels on trigger day (E2 ≥ 18350 pmol/ml) - 0.092 * P levels on trigger day + 0.0144 * BMI + 0.0028 * AMH))).
The 3rd phase: P (OHSS) = 1 / (1 + exp(- (- 4.7715 - 0.69 * Number of COS cycles - 0.2899 * Previous live birth (1) + 1.057 * Body surface area - 0.0173 * AFC + 0.4284 * PCOS (yes) -0.27 * GnRH antagonist protocol - 1.6428 * Freeze-all (yes) - 0.1359 * Duration of Gn - 0.0061 * Mean Dose of Gn - 0.0067 * Initial dose of Gn - 2.1356 * Trigger type (GnRHa alone) + 1.9909 * Trigger type (HCG alone) + 0.026 * Number of follicles (≥ 10 mm) on trigger day + 0.0947 * Number of follicles (≥ 18 mm) on trigger day + 0.9162 * E2 levels on trigger day (3670 ≤ E2 < 18350 pmol/ml) + 1.391 * E2 levels on trigger day (E2 ≥ 18350 pmol/ml) + 0.0328 * Number of oocytes retrieved))).
The 4th phase: P (OHSS) = 1 / (1 + exp(- (- 5.5972 - 0.5855 * Number of COS cycles -0.3028 * Previous live birth (1) + 0.9797 * Body surface area - 0.0173 * AFC + 0.4322 * PCOS (yes) - 0.7264 * Freeze-all (yes) - 0.1045 * Duration of Gn - 0.0069 * Mean dose of Gn - 0.0065 * Initial dose of Gn - 2.07 * Trigger type (GnRHa alone) + 1.9291 * Trigger type (HCG alone) + 0.0892 * Number of follicles ( 16 mm) on trigger day + 0.6846 * E2 levels on trigger day (3670 ≤ E2 < 18350 pmol/ml) + 1.5483 * E2 levels on trigger day (E2 ≥ 18350 pmol/ml) + 0.0562 * Number of oocytes retrieved + 0.2702 * Number of cycles with ET + 0.5219 * Number of embryo(s) for ET + 0.1745 * Number of top-quality embryo(s) for ET))).

### Performance of the multiphase prediction models

**Figure 2:**
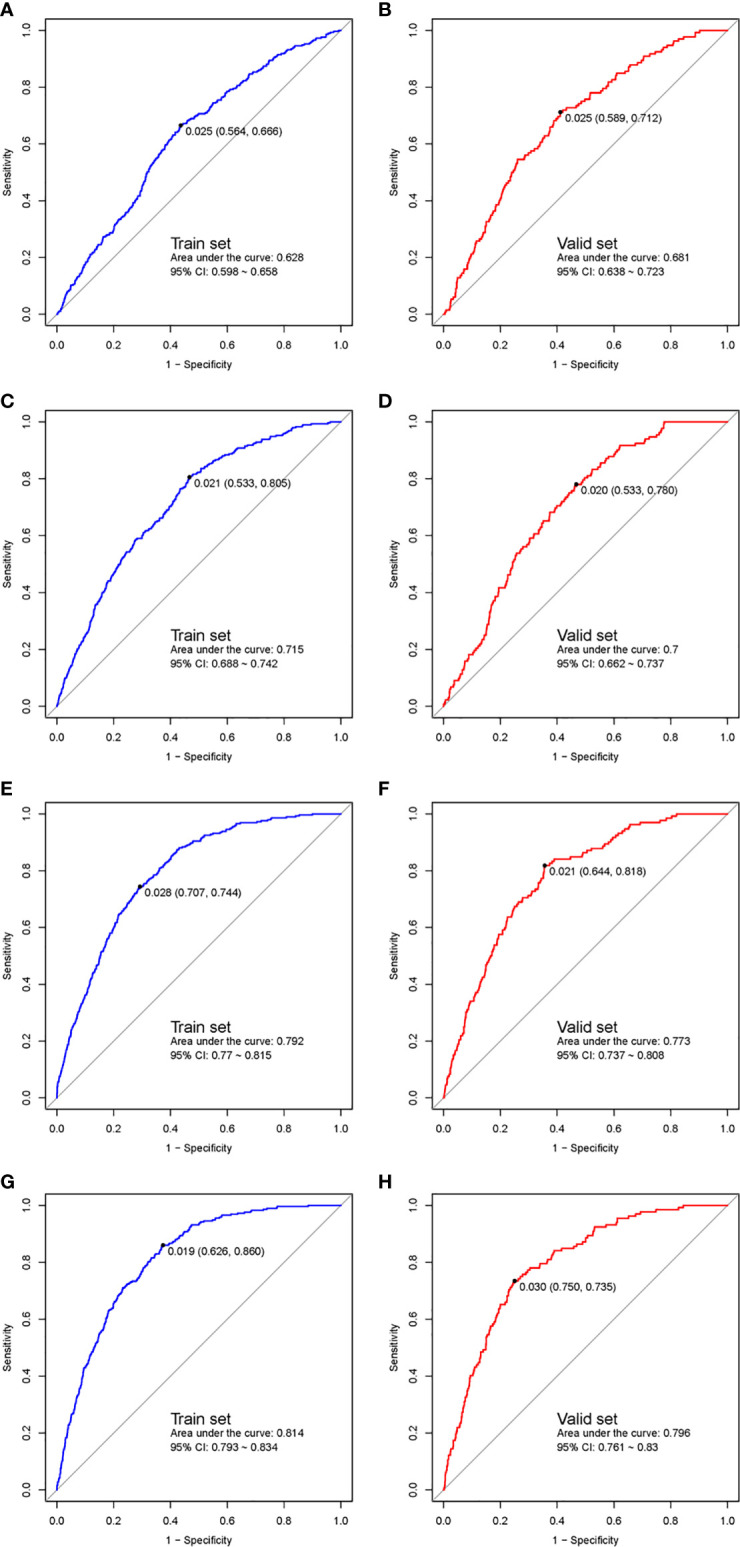
ROC curves of the multiphase prediction models in the training cohort (left panel) and validation cohort (right panel). The first phase (training cohort, **A**; validation cohort, **B**), the 2nd phase (training cohort, **C**; validation cohort, **D**), the 3rd phase (training cohort, **E**; validation cohort, **F**), and the fourth phase (training cohort, **G**; validation cohort, **H**). ROC, receiver-operator characteristic.

ROC curves of both the training cohort and validation cohort were established and are demonstrated in **Figure 2**. The AUC or C-statistic in the training cohort in the four phases were 0.628 (95% CI 0.598–0.658, **Figure 2A**), 0.715 (95% CI 0.688–0.742, **Figure 2C**), 0.792 (95% CI 0.770–0.815, **Figure 2E**), and 0.814 (95% CI 0.793–0.834, **Figure 2G**), respectively. The C-statistics in the validation cohort in the four phases were 0.681 (95% CI 0.638–0.723, **Figure 2B**), 0.700 (95% CI 0.662–0.737, **Figure 2D**), 0.773 (95% CI 0.737–0.808, **Figure 2F**), and 0.796 (95% CI 0.761–0.830, **Figure 2H**), respectively.

**Figure 3:**
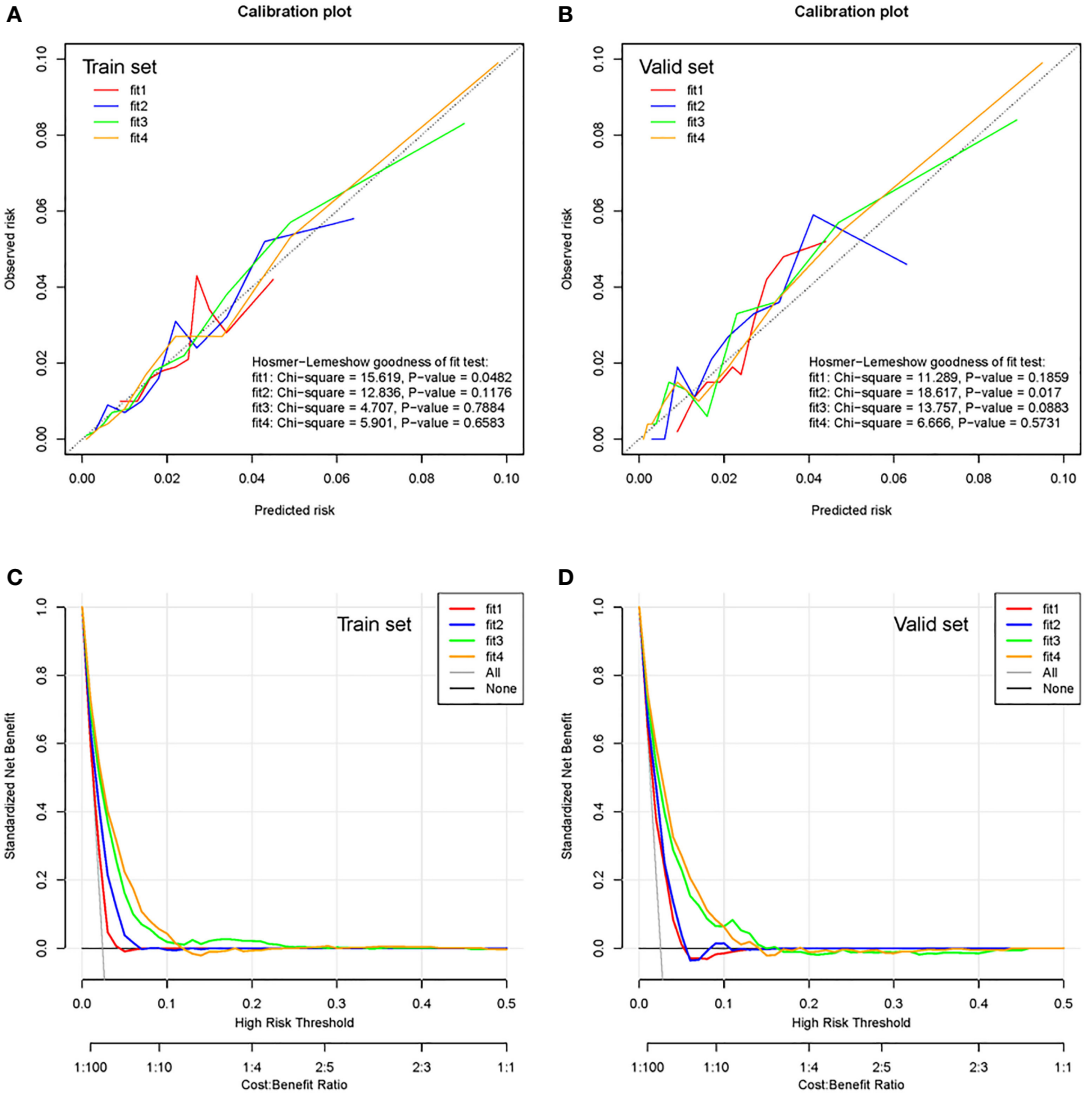
Calibration plots and clinical decision curves of the training cohort and validation cohort. Calibration plots of the multiphase prediction models in the training cohort **(A)** and validation cohort **(B)**. Clinical decision curves of the multiphase models in the training cohort **(C)** and validation cohort **(D)**. Fit 1 represents the first-phase prediction model, fit 2 represents the second-phase prediction model, fit 3 represents the third-phase prediction model, and fit 4 represents the fourth-phase prediction model.

The calibration plot (**Figure 3A** for the training cohort and **3B** for the validation cohort) showed high calibration for the third- and fourth-phase prediction models in both the training and validation cohorts. The third- and fourth-phase prediction models also showed the highest net benefit (**Figure 3C** for the training cohort and **3D** for the validation cohort).

The OHSS discrimination abilities of the multiphase models in normal responders and hyper-responders were also assessed and presented in Supplemental data (**Supplementary Figure 2** for normal responders, and **Supplementary Figure 3** for hyper-responders). As expected, the prediction models were reliable in predicting the OHSS in either normal responders or hyper-responders.

### Construction of the smartphone-based app

**Figure 4:**
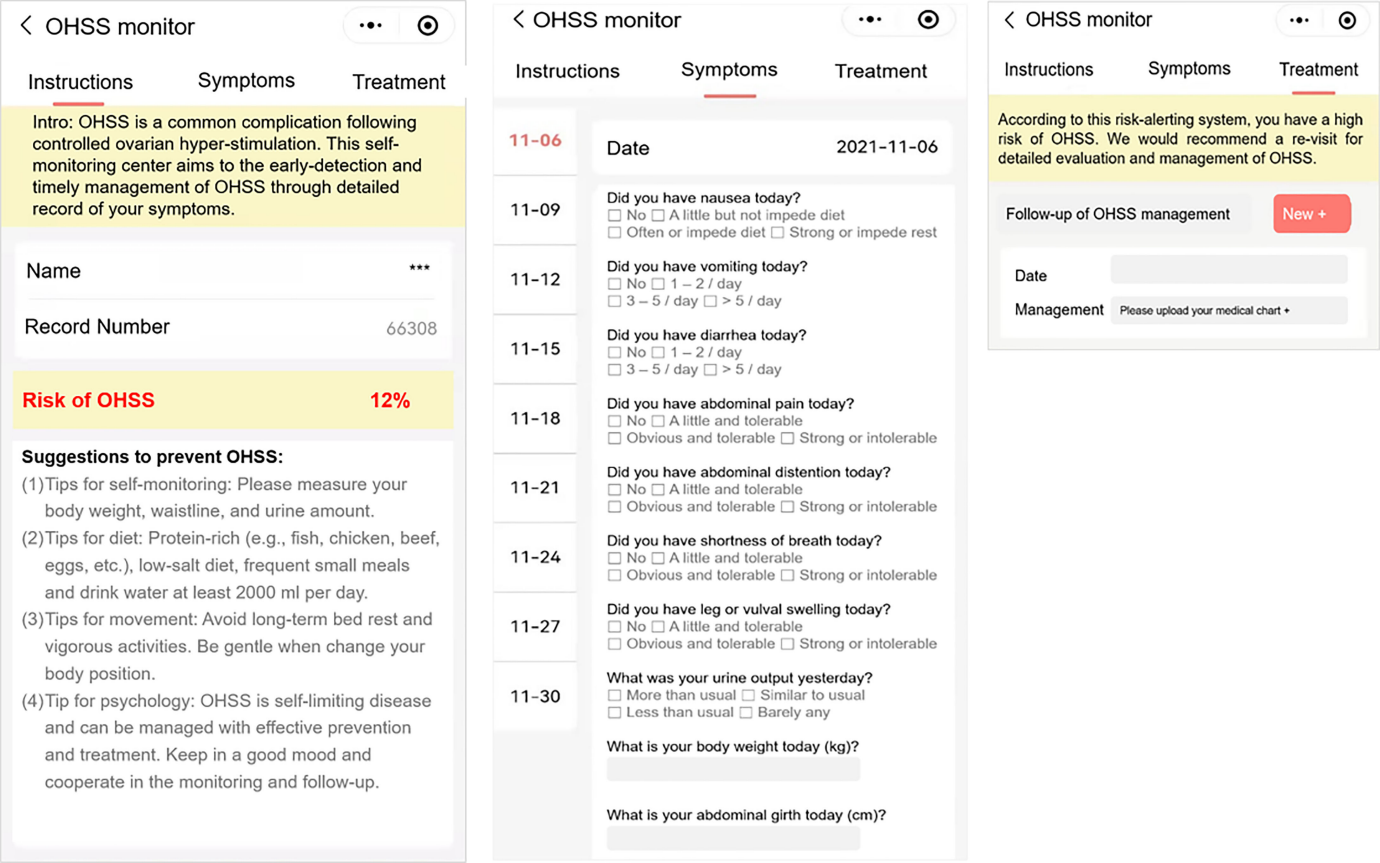
Screenshot of the “OHSS monitor” app from a patient’s view.

The smartphone-based app named “OHSS monitoring” has been constructed and built in the routinely used hospital database system which was designed for women during the ART treatment procedures, with full access to clinical physicians and partial access to ART patients. The app can provide a predictive risk of OHSS at four different phases of the COS cycle and help to support clinical decision-making (the first to third phases can only be accessed with clinical physicians as a “risk calculator”) and self-monitoring (the fourth phase, accessed with both clinical physicians and OHSS at-risk patients). A brief demo of the app for patients’ self-monitoring is shown in **Figure 4**.

## Discussion

Here, based on a retrospective cohort of more than 21,000 women from a high-volume reproductive center, we generated quantified and complex multiphase prediction models of OHSS with a smartphone-based user-friendly app for clinicians’ risk calculation and patients’ self-monitoring. These multiphase prediction models can accurately distinguish women who may develop moderate/severe OHSS from those who would not. The predicted and observed risks of OHSS were concordant in the multiphase prediction models. Besides, the multiphase prediction models would be favorable in clinical utility with high net benefit. The multiphase prediction models can be used for the unselected population in the reproductive center with satisfactory discrimination and calibration. The multiphase prediction and self-monitoring app can be readily used in clinical settings.

Several prediction models of OHSS have been reported (9). One particular predictive model recruited PCOS women exclusively (10) and provided a prediction of OHSS after oocyte retrieval, whereas an early prediction prior to oocyte retrieval might be more necessary since physicians can rearrange the ovarian stimulation protocols and ovulation trigger options accordingly. Moreover, the reported prediction model included PCOS patients only with fresh embryo transfer, which might neglect the occurrence of OHSS in the population with all embryos cryopreserved. Other models reported either limited to a couple of selected parameters for prediction (11, 12), or restricted with a certain population (for instance, PCOS population only (10, 13), non-PCOS patients with long GnRH agonist protocol (14), patients with GnRH agonist protocol and HCG for triggering (9), coasted patients in the late follicular phase with long GnRH agonist protocol (15). Besides, the majority of the available predictions were established based on a relatively small sample size. Unlike those previously reported prediction models, multiphase prediction models in the present study focused more on the unselected general population and covered the whole spectrum of the COS process from the initiation of COS to fresh embryo transfer.

The prediction of OHSS has not been considered as an easy task. One main reason is that tons of influencing factors are contributing to the cascade of events leading to OHSS. Several ovarian reservation markers, including age, AFC, and AMH, have been thoroughly invested for their value in predicting OHSS (4, 16). Generally, it is believed that young and slim women with increased ovarian reserve were high-risk populations to develop OHSS (2). It is interesting to detect an association of low dose or duration of gonadotropin usage and increased risk of OHSS in the multiphase prediction models. Although no exact mechanisms were confirmed, the higher risk of OHSS might be due to the improved sensitivity to gonadotropin. To be particular, those hyper-responders of ovarian stimulation would require a low dose of gonadotropin and still possess a higher risk of developing OHSS (17). Elevated E2 levels were well-documented as an etiological factor of OHSS (18). Sustained supraphysiological levels of E2 might be associated with elevated number of oocytes retrieved as well as number of usable embryos (19). It is reasonable that increased E2 levels also contributed to the development of OHSS as indicated in the multiphase prediction models. Results from the multiphase prediction models also showed a similar trend of such featured women with a higher risk of developing OHSS.

It is not surprising that GnRHa for trigger and freeze-all strategy are major indicators involved in the prediction models (20). A single dose of GnRHa for trigger has also been shown to be a highly effective way for OHSS prevention in GnRH antagonist protocol (2). Plenty of evidence has confirmed that GnRHa trigger can notably reduce and even eliminate the occurrence of OHSS (3, 21, 22) Elective cryopreservation of all embryos or freeze-all strategy has been well known for preventing OHSS (2, 22, 23). The secretion of endogenous HCG along with pregnancy contributes to the exacerbation of OHSS symptoms and leads to the late onset of OHSS. Therefore, with a freeze-all strategy, the late onset of OHSS can largely be avoided (2). Even though few cases of OHSS occurred following freeze-all, a reduced severity of OHSS symptoms was observed (24). Undoubtedly, an increased number of follicles on trigger day and an elevated number of oocytes collected would promote the development of OHSS and can be useful in OHSS prediction (25). Fresh embryo transfer was one important trigger for late-onset OHSS. As expected, patients with embryo transfer, especially top-quality embryos, would have a higher risk of OHSS as demonstrated by the multiphase prediction models.

As an iatrogenic complication, the occurrence of OHSS is comprehensive result of patients’ characteristics and physicians’ interventions. Therefore, the first-phase prediction model, which was largely based on patients’ baseline clinical features, showed the lowest ability to discriminate OHSS from the non-OHSS population, whereas the prediction models of the other three phases all demonstrated to have reasonable performances. Besides, the C-statistics increased along with the process of the phases, as the acceptable c-statistic should be at least more than 0.7 (7). This low accuracy of very early phase prediction is probably due to multiple preventive or promotive interventions provided during the following COS process, which might cause a deviation in the prediction of occurrence of OHSS. For instance, it is commonly believed that women with significantly higher AMH levels and low BMI were more likely to develop OHSS. However, for those natively “high-risk” women, several prevention approaches might have already been provided by experienced physicians. In this case, those “high-risk” women might eventually be “low-risk” women for developing OHSS. Given the complexity of assisted reproductive technologies and the human reproduction process, the C-statistics above 0.6 are acceptable in clinical practice (26), and the accuracies of prediction models could improve with more variables introduced into the formulas.

Apps are an ideal tool to transfer the complex nomograms to a simple risk assessment result and can be easily applied in clinical practice. The majority of medical apps available are designed for healthcare information provision. Predictive-modeling-based medical apps only represented 16% of clinical decision support modality (27). Here, we developed a predictive-model-based app from rigorous data analysis of a large database. One major advantage of this predictive-modeling-based app is the individualized risk assessment provided for specific women at specific time points of consultation. Thus, an individualized clinical decision could be made accordingly. Especially, since assisted reproductive care is only available in almost urban areas only, women in low-income settings who do not have easy access to healthcare can particularly benefit from this smartphone-based app to have an early alert of OHSS risk. By automatically collecting values of parameters from the clinical database, the app would indicate an individualized risk score. Based on the predictive risk, physicians can choose an optimal strategy to lower the OHSS risk in the following process. A strict follow-up and careful self-monitoring would be suggested for those at risk of developing OHSS. Taking advantage of the self-monitoring module of the app, patients would be more aware of OHSS-related symptoms, for example, nausea, abdominal distention, and abdominal pain, and make revisit schedules once improved risks of OHSS are alerted by the app. With the help of the self-monitoring module, delayed diagnosis and management of OHSS can be largely reduced and even eliminated. This clinical decision-support and self-monitoring app has tremendous potential to improve the management of OHSS-risky women and enhance the safety of ART, although a prospective study is needed in the future to confirm the performance of this app tool.

### Clinical Implications

**Figure 5:**
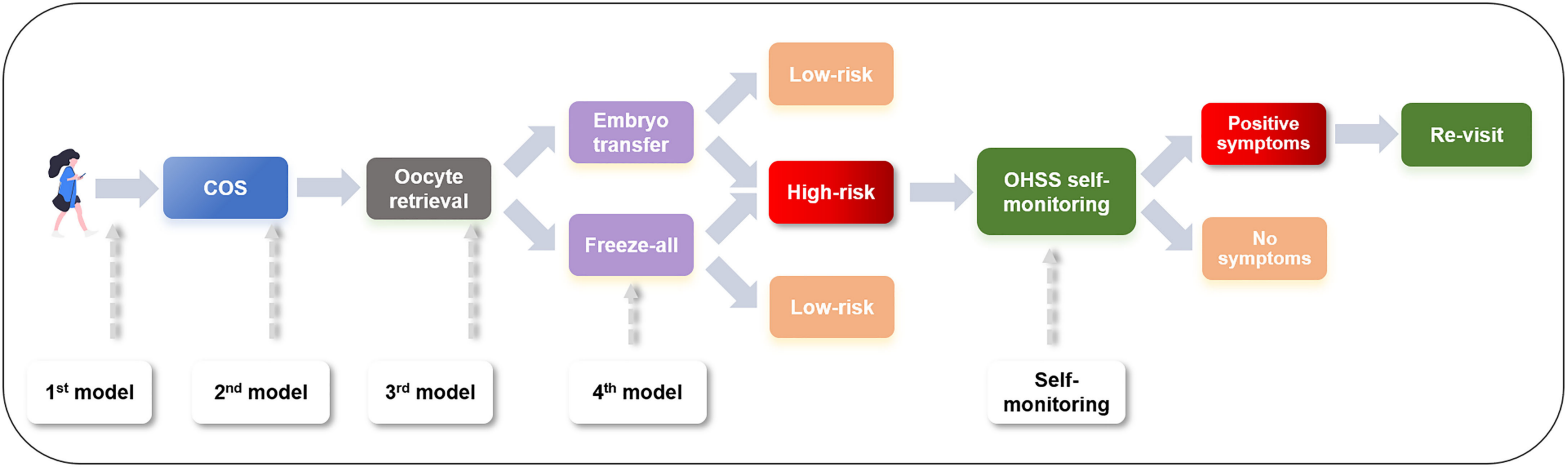
Workflow of management of an OHSS patient. OHSS, ovarian hyper-stimulation syndrome; COS, controlled ovarian stimulation.

The proposal of multiphase prediction models of OHSS is particularly important in practical terms. Stratification based on the risk ratio and workflow of women with different risk potentials are summarized in **Figure 5**. It is of great clinical value to predict OHSS before the initiation of COS based on baseline clinical features; hence, clinicians can provide treatment options to prevent OHSS. During the COS and even after oocyte retrieval, with the availability of more parameters, the assessment of OHSS becomes more accurate, which allows clinicians to provide preventive approaches beforehand. For clinicians, with the indication of individualized OHSS at-risk women, several preventive clinical interventions, including COS protocol chosen, GnRHa triggering, and freeze-all strategy, can be applied appropriately. For at-risk patients, the implementation of this user-friendly app could enhance patients’ access to healthcare of OHSS and improve the quality of care, especially for patients in remote areas. An accurate prediction of OHSS could minimize unnecessary interventions, for instance, repeated revisit, and provide remarkable benefits for both clinicians and patients.

### Strengths and Limitations

This study has evaluated more than 21,000 women and produced big data-based multiphase prediction models for OHSS in the general population with satisfactory accuracies. Right now, multifunction app development in the healthcare field is still in its infancy. Here we present one of the few apps reported in ART with predictive-model-based clinical decision-supporting and self-management function. Comprehensive risk assessment, individualized decision-making, and self-monitoring for at-risk individuals could benefit from this predictive-model-based app. However, a few limitations of this multiphase model are worth mentioning. First of all, the present study did not include mild OHSS, since the retrospective design of this study cannot collect detailed information of women with mild OHSS. Secondly, early onset and late onset of OHSS were not specified in the present study, which should be reevaluated in our prospective study. Moreover, due to the retrospective nature of the study design, a couple of parameters, for example, the doses of HCG for triggering, coasting, and other measures attempting to eliminate OHSS symptoms, were not included in the present models. To be a useful tool in daily practice, the predictive ability of the multiphase model and prediction-model-based app should be further assessed in prospective clinical trials to evaluate their cost-effectiveness, safety, and benefit in real-world settings.

## Conclusion

This study developed multiphase prediction models of OHSS based on retrospective big data, to help in the evaluation and decision-making of physicians, which can reliably distinguish patients with a high risk of OHSS. By empowering high-risk patients with the smart-phone based self-monitoring app, early detection and management of OHSS can be easily implemented. The management strategies of patients with a high risk of OHSS would be improved with the help of multiphase prediction models and a predictive-model-based app. A combination of the OHSS prediction model and app could serve as efficient support for personalized management of OHSS. Ongoing prospective validations of the predictive model and the app are performed to further evaluate their clinical utility.

## Data Availability Statement

The data underlying this article will be shared on reasonable request to the corresponding authors.

## Ethics Statement

The studies involving human participants were reviewed and approved by the Ethical Committee of the Third Affiliated Hospital of Guangzhou Medical University (approval number, 2021-117). The patients/participants provided their written informed consent to participate in this study.

## Author Contributions

MC, ZL, and YLin contributed to the study design, data acquisition, data analysis, and manuscript draft writing. YLuo, SL, and QH contributed to the data acquisition and data interpretation. HL and JL contributed to the study design, data interpretation, and draft revision. All authors contributed to the article and approved the submitted version.

## Funding

This study was supported by grants from the Science and Technology Program of Guangzhou, China (No. 202102010076, for data acquisition), and The Medical Key Discipline of Guangzhou (2021-2023, for data analysis).

## Conflict of Interest

The authors declare that the research was conducted in the absence of any commercial or financial relationships that could be construed as a potential conflict of interest.

## Publisher’s Note

All claims expressed in this article are solely those of the authors and do not necessarily represent those of their affiliated organizations, or those of the publisher, the editors and the reviewers. Any product that may be evaluated in this article, or claim that may be made by its manufacturer, is not guaranteed or endorsed by the publisher.

## Supplementary Material

Supplementary Figure 1 Nomograms of the multi-phase prediction models to predict OHSS in the general population. Nomograms of the 1^st^ phase (2A), the 2^nd^ phase (2B), the 3^rd^ phase (2C), and the 4^th^ phase (2D). Abbreviations: OHSS, ovarian hyper-stimulation syndrome; COS, controlled ovarian stimulation; AMH, anti-Mullerian hormone; BMI, body mass index; PCOS, polycystic ovarian syndrome; Gn, gonadotropin; E2, estradiol; P progesterone; AFC, antral follicle counting; ET, embryo transfer.

Supplementary Figure 2 ROC curves of the multi-phase prediction models in the training cohort (left panel) and validation cohort (right panel) in normal-responders. The 1^st^ phase (training cohort, **Supplementary 1A**; validation cohort, **Supplementary 1B**), the 2^nd^ phase (training cohort, **Supplementary 1C**; validation cohort, Supplementary 1D), the 3^rd^ phase (training cohort, **Supplementary 1E**; validation cohort, **Supplementary 1F**), and the 4^th^ phase (training cohort, **Supplementary 1G**; validation cohort, **Supplementary 1H**).

Supplementary Figure 3 ROC curves of the multi-phase prediction models in the training cohort (left panel) and validation cohort (right panel) in hyper-responders. The 1^st^ phase (training cohort, **Supplementary 2A**; validation cohort, **Supplementary 2B**), the 2^nd^ phase (training cohort, **Supplementary 2C**; validation cohort, **Supplementary 2D**), the 3^rd^ phase (training cohort, **Supplementary 2E**; validation cohort, **Supplementary 2F**), and the 4^th^ phase (training cohort, **Supplementary 2G**; validation cohort, **Supplementary 2H**).
