# Supplementary material for: A Personalized Management Approach of OHSS: Development of a Multiphase Prediction Model and Smartphone-Based App
Source: Front Endocrinol (Lausanne). 2022 Jul 6;13:911225. doi: 10.3389/fendo.2022.911225 (PMC9296830; doi:10.3389/fendo.2022.911225)

**Supplemental Figure 1.** Nomograms of the multi-phase prediction models to predict OHSS in the general population.

**
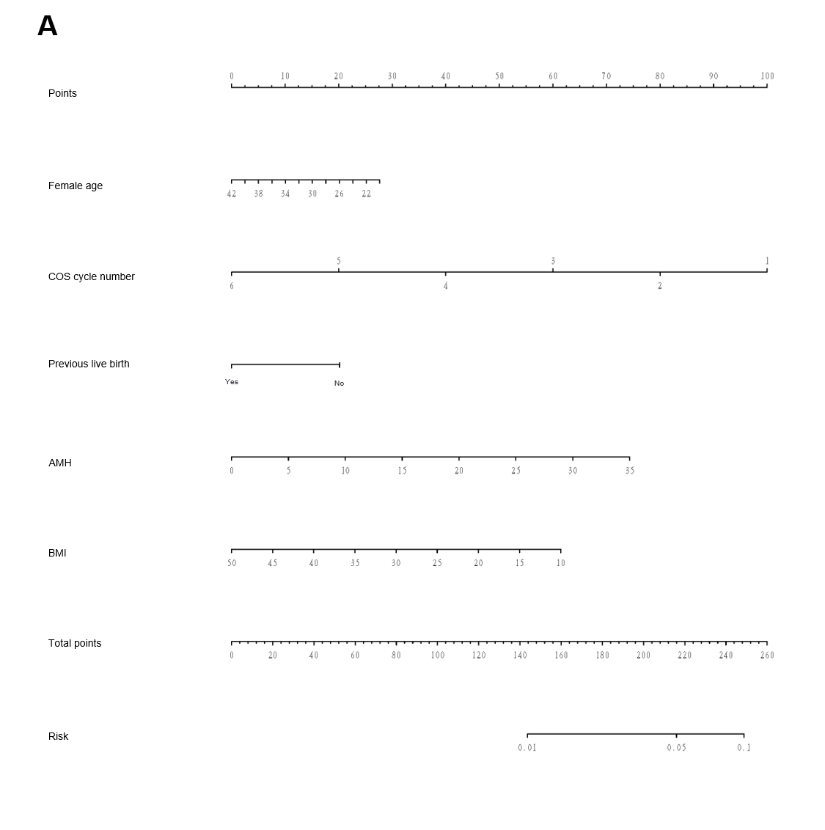

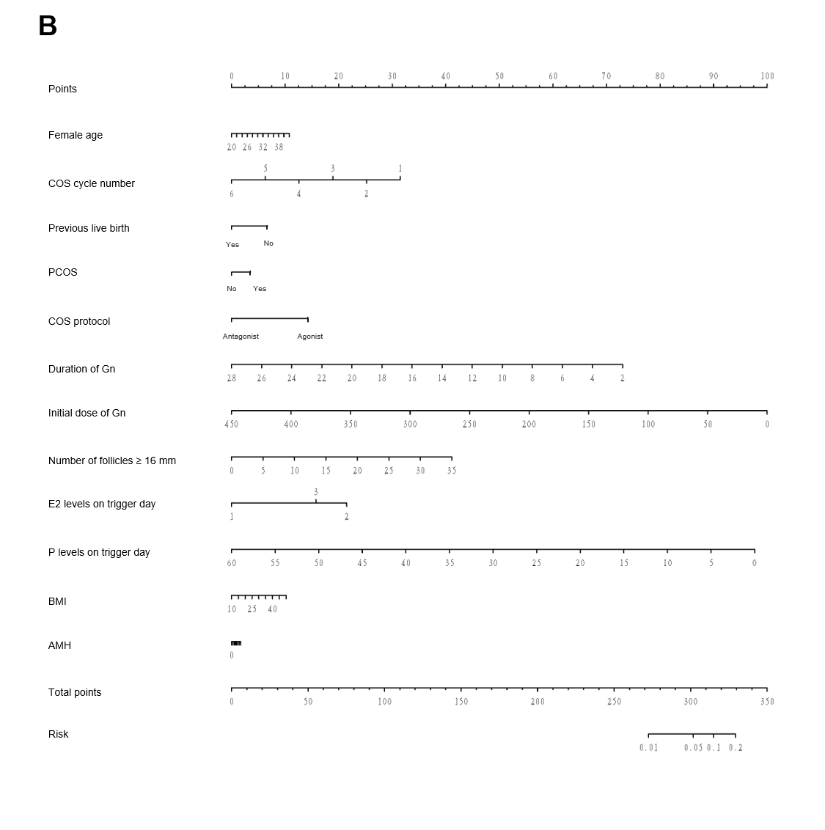

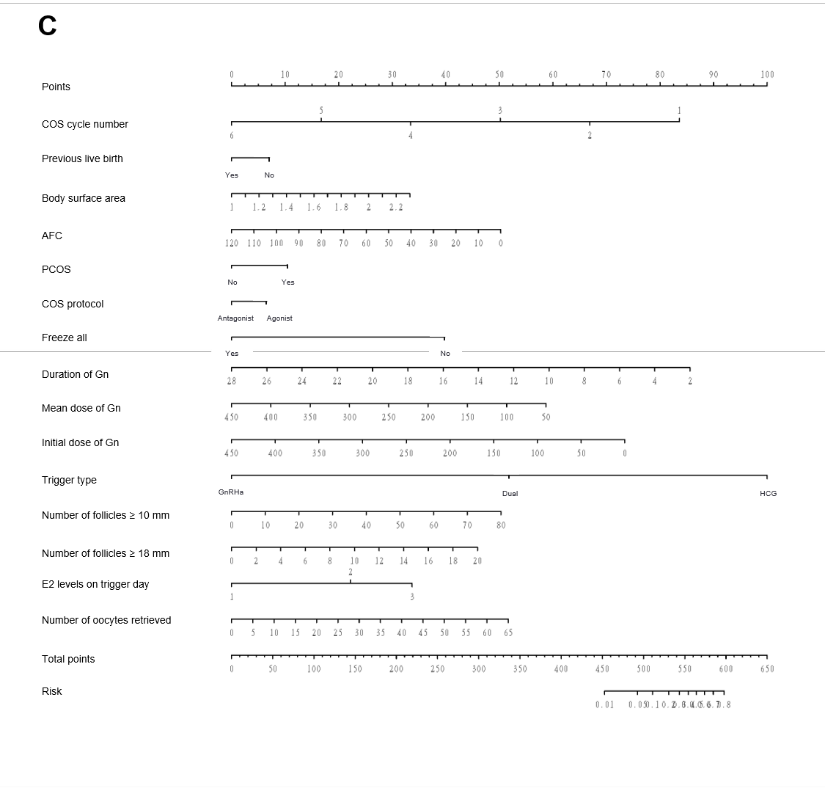

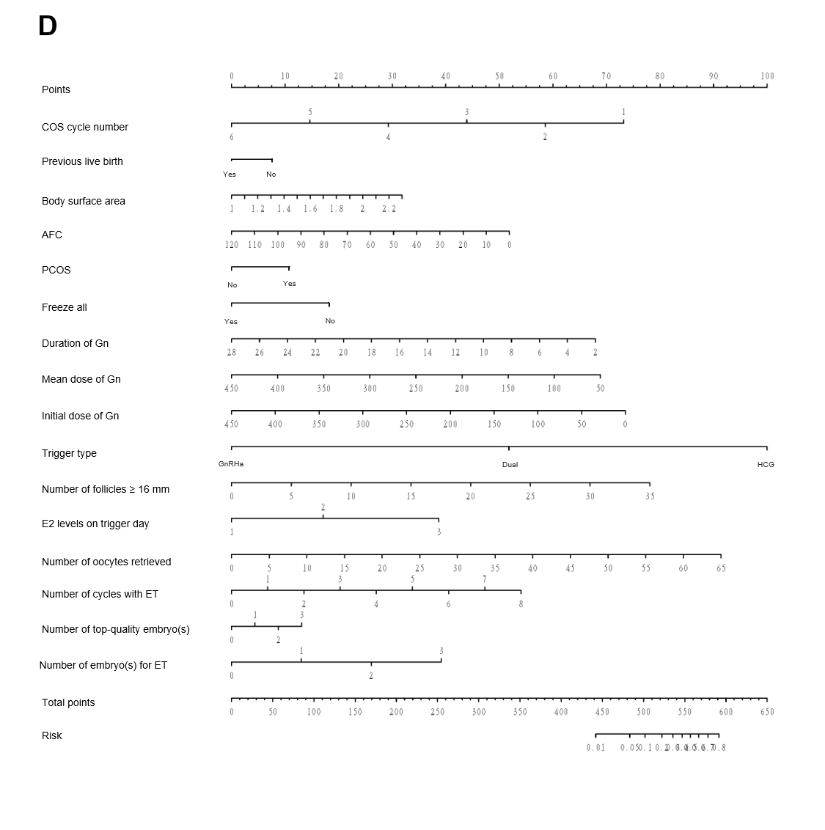
**

**Supplemental Figure 2.** ROC curves of the multi-phase prediction models in the training cohort (left panel) and validation cohort (right panel) in normal-responders.


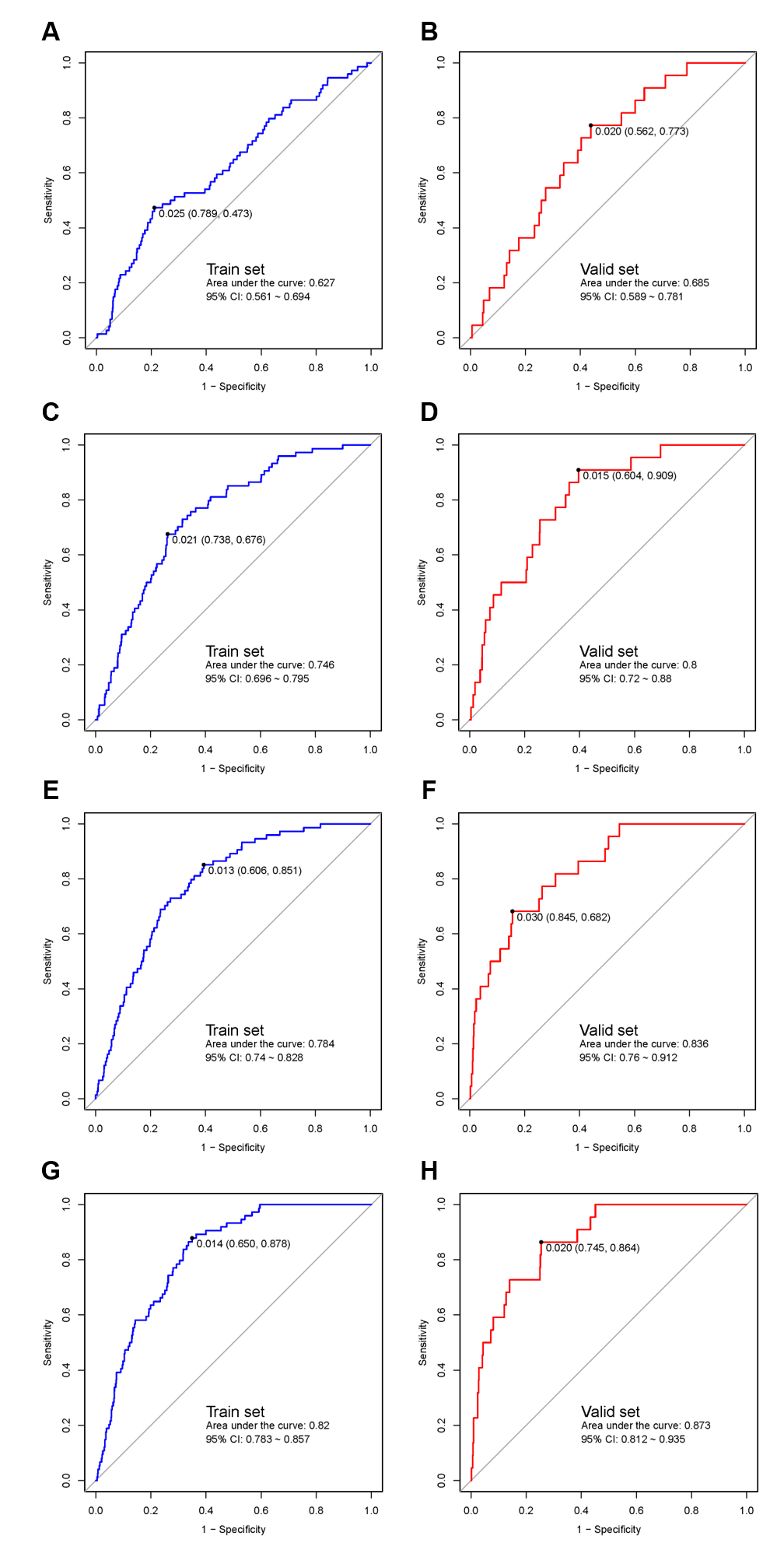


**Supplemental Figure 3.** ROC curves of the multi-phase prediction models in the training cohort (left panel) and validation cohort (right panel) in hyper-responders.


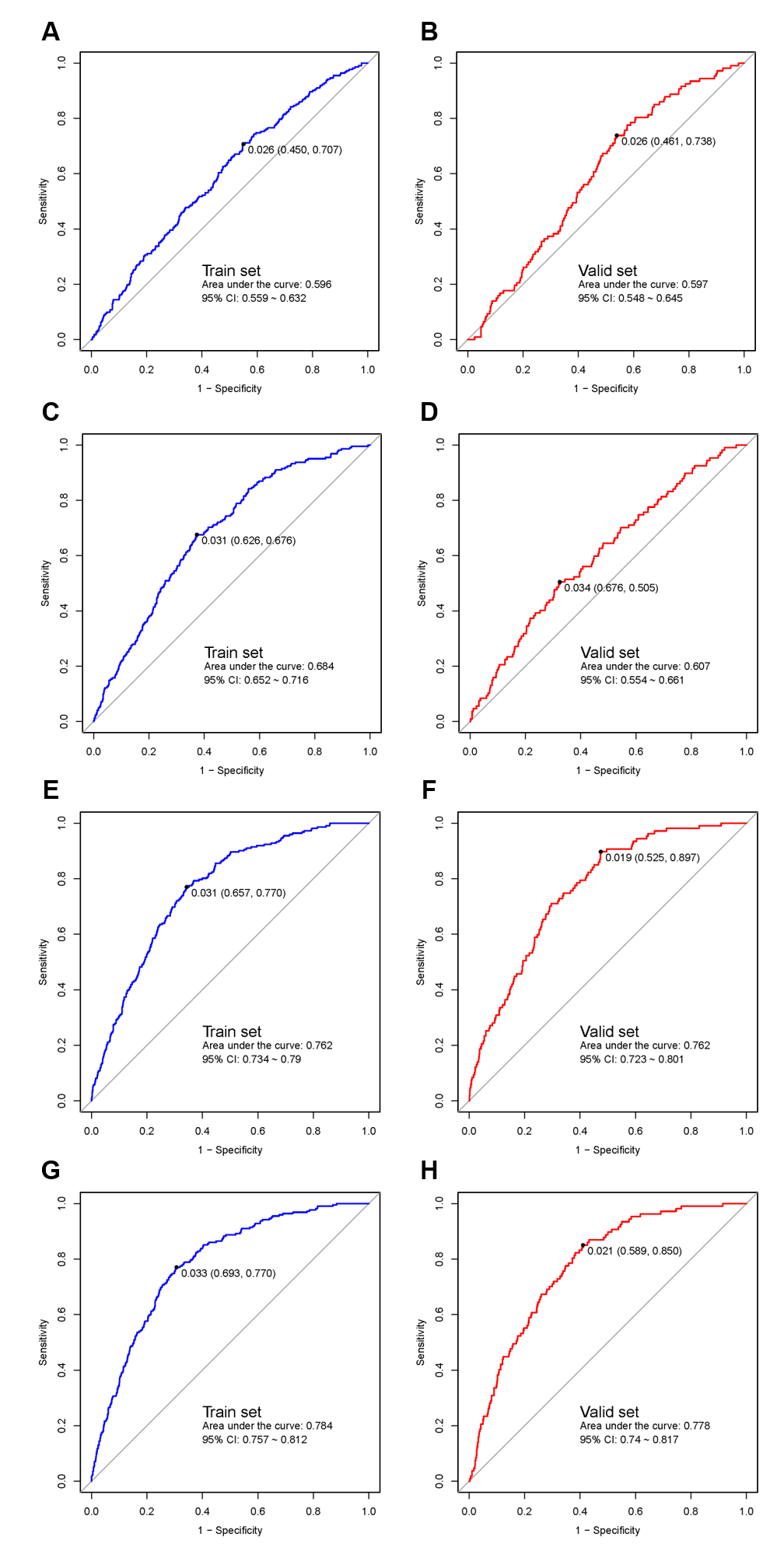

Supplement: Supplementary Figure 1 — Nomograms of the multi-phase prediction models to predict OHSS in the general population. Nomograms of the 1st phase (2A), the 2nd phase (2B), the 3rd phase (2C), and the 4th phase (2D). Abbreviations: OHSS, ovarian hyper-stimulation syndrome; COS, controlled ovarian stimulation; AMH, anti-Mullerian hormone; BMI, body mass index; PCOS, polycystic ovarian syndrome; Gn, gonadotropin; E2, estradiol; P progesterone; AFC, antral follicle counting; ET, embryo transfer. [file DataSheet_1.docx]
